# Exploration du grêle par entéroscopie simple ballon, expérience de l´Hôpital militaire et d´instruction Mohamed V de Rabat: à propos de 51 cas

**DOI:** 10.11604/pamj.2021.39.61.29200

**Published:** 2021-05-21

**Authors:** Mainkouado Michael Samy, Rachid Laroussi, Tarik Adioui, Hassan Seddik

**Affiliations:** 1 Service Gastroentérologie, Hôpital Militaire Instruction Mohamed V, Rabat, Maroc

**Keywords:** Entéroscopie simple ballon, faisabilité, rentabilité diagnostique, rentabilité thérapeutique, Single balloon enteroscopy, feasibility, diagnostic efficiency, therapeutic efficiency

## Abstract

L´entéroscopie est devenue une technique incontournable dans l´exploration mais surtout dans le traitement des lésions de l´intestin grêle. Elle est en général réalisée suite à un examen préalable du grêle par vidéo-capsule endoscopique. Trois techniques équivalentes existent: l´entéroscopie double ballon, l´entéroscopie simple ballon et l´entéroscopie spiralée. Le but de cette étude est de décrire la faisabilité technique de l´entéroscopie simple ballon ainsi que sa tolérance, ses indications et ses résultats obtenus dans notre expérience. Une analyse rétrospective et descriptive s´étalant sur 8 ans des dossiers de patients ayant bénéficié d´une entéroscopie simple ballon au sein du service de gastro-entérologie de l´hôpital militaire et d´instruction Mohamed V de Rabat. Les critères d´inclusion étaient: être atteint ou suspecté d´une pathologie du grêle soit à l´imagerie soit à la vidéo-capsule-endoscopique et avoir bénéficié d´une entéroscopie simple ballon. Les variables étudiées étaient la faisabilité technique du geste, ses indications, ses résultats ainsi que ses complications. Cinquante-un (51) patients dont 30 hommes et 21 femmes d´âge moyen 48 ans (18 ans-91 ans) ont été inclus dans l´étude. La technique adoptée chez tous les patients était une entéroscopie simple ballon sous anesthésie générale avec intubation des voies respiratoires. La durée de l´examen était en moyenne de 45 min par voie haute et de 60 min par voie basse. Le grêle a été exploré jusqu´à l´iléon proximal en cas d´entéroscopie haute et jusqu´au-delà de 120cm de la DAI en cas d´entéroscopie basse. Les indications étaient représentées par les saignements digestifs inexpliqués (72%), épaississements grêliques (17%), suspicion de tumeur grêlique (6%), bilan d´évaluation d´un crohn grêlique (4%), et retrait de la vidéo-capsule endoscopique (VCE) (2%). Un diagnostic a été réalisé ou confirmé chez 29 patients soit une rentabilité diagnostic de 57%. Les angiodysplasies représentaient 70% des lésions retrouvées, les ulcérations grêliques (10%), les sténoses grêlique (7%), les tumeurs grêliques (7%), des diverticules grêliques (3%) et un retrait spontané de la VCE (3%). Un traitement endoscopique a été possible chez 20 patients soit une rentabilité thérapeutique de 39%; il s´agissait d´une électrocoagulation au plasma argon des lésions d´angiodysplasies grêliques. Aucune complication n´a été observée dans notre série. L´entéroscopie simple ballon reste donc un examen bien toléré qui permet d´explorer une grande longueur du grêle. Ses indications étaient nombreuses et variées dans notre étude et ses rentabilités diagnostic et thérapeutique étaient satisfaisantes.

## Abstract (en)

Enteroscopy has become an indispensable technique for the exploration and, in particular, the treatment of small intestine lesions. It is usually performed following video capsule endoscopy of the small intestine. Three equivalent techniques exist: double balloon enteroscopy, single balloon enteroscopy and spiral enteroscopy. The purpose of this study is to describe the technical feasibility of single balloon enteroscopy as well as its tolerance, indications and the results obtained in our context. We conducted a retrospective and descriptive analysis of the records of patients undergoing single balloon enteroscopy in the Department of Gastroenterology at the Mohammed V Training Military Hospital in Rabat over an 8-year period. Inclusion criteria were: small intestine disease or the suspicion of small intestine disease on imaging tests or video-capsule endoscopy and having undergone single balloon enteroscopy. The variables studied were the technical feasibility of single balloon enteroscopy, its indications, results and complications. Fifty-one (51) patients, including 30 men and 21 women with an average age of 48 years (18 years-91 years), were included in the study. The technique used in all patients was single balloon enteroscopy under general anesthesia with intubation. The mean duration of single balloon enteroscopy via the upper gastrointestinal (GI) tract (antegrade) was 45 min and 60 min via the lower GI tract (retrograde). The small intestine was explored up to the proximal ileum via the upper gastrointestinal (GI) tract and through more than 120cm from IAD via the lower GI tract. The indications included: unexplained digestive bleeding (72%), small intestine wall thickening (17%), the suspicion of small intestine tumor (6%), evaluation of Crohn´s disease (4%) and endoscopic video capsule (ECV) reduction (2%). Diagnosis was made or confirmed in 29 patients, reflecting a diagnostic efficiency of 57%. Angiodysplasia accounted for 70% of the lesions, ulcerations 10%, stenoses 7%, tumors 7%, diverticula 3% and spontaneous ECV reduction 3%. Endoscopic treatment was performed in 20 patients, with a therapeutic efficiency of 39%; it was based on argon plasma coagulation of gastroenteric angiodysplasia lesions. No complications were observed in our case series. Single balloon enteroscopy is a well-tolerated examination which allows for the exploration of a long portion of the small intestine. Multiple and various indications were provided in our study and the diagnostic and therapeutic benefits were satisfactory.

## Introduction

**Figure 1:**
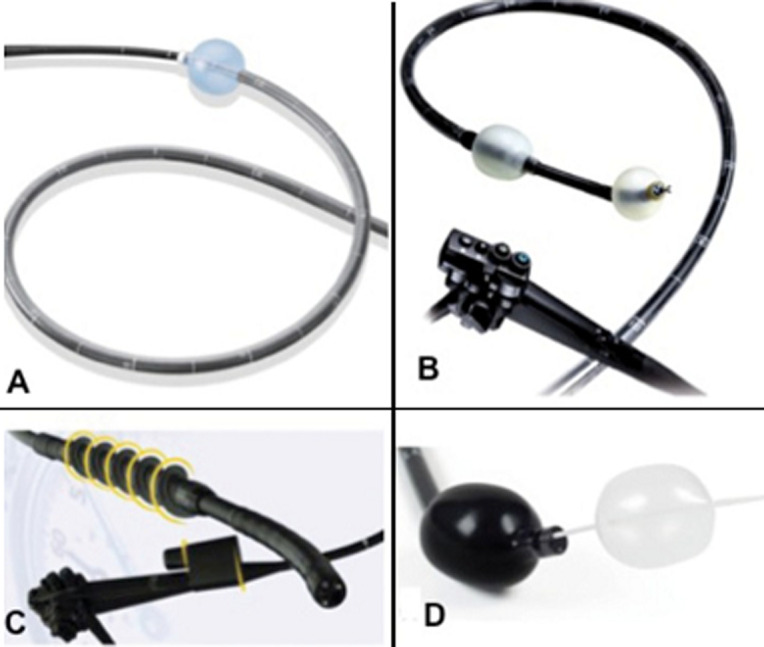
les différents systèmes d´entéroscopie; simple ballon (A), double ballon (B), entéroscopie spiralée motorisée (C) et l´entéroscopie avec le système de ballon G-EYE (D)

L´intestin grêle est la partie de l´appareil digestif humain située entre l´estomac et le gros intestin (colon). Sa longueur moyenne est 6 m et peut varier de 4 à 7 mètres, selon la technique de mesure utilisée. Ses différentes parties anatomiques sont le duodénum (0,25 m), le jéjunum (2,5 m), et l´iléon (3,5 m). Il joue le rôle majeur de la fonction d´absorption. L´intestin grêle est un organe complexe à explorer, du fait de sa longueur. A ce jour ses moyens d´explorations sont: 1) le cliché d´abdomen sans préparation, réalisé debout et de face, est un examen de débrouillage recherchant principalement des niveaux hydro-aériques, signes d´occlusion. 2) L´entéroscanner et l´entéro-imagerie par résonance magnétique (IRM) grâce à l´ingestion d´un produit de contraste (respectivement la baryte et le gadolinium) permettent de visualiser des zones sténotiques, notamment dans la maladie de Crohn. 3) La fibroscopie œsogastroduodénale explore le duodénum jusqu´à l´angle de Treitz, et la coloscopie peut explorer la dernière anse grêlique. 4) La vidéo-capsule endoscopique (VCE) est la seule procédée permettant l´exploration complète de l´intestin grêle, analyse et prises de photos par micro-caméra embarquée dans une capsule étanche; 5) et enfin l´entéroscopie. Cette dernière est devenue un examen incontournable dans l´exploration mais surtout dans le traitement des lésions de l´intestin grêle. Elle est en général réalisée suite à un examen préalable du grêle par vidéo-capsule endoscopique Comme illustré sur la Figure 1, quatre variétés d´entéroscopie ont été développées [1-4]: l´entéroscopie avec le système de ballon G-EYE, l´entéroscopie spiralée , l´entéroscopie double ballon et l´entéroscopie simple ballon. Le but de cette étude est de décrire la faisabilité, la tolérance ainsi que les indications et les résultats de l´entéroscopie simple ballon obtenus dans un groupe de patients atteints ou suspects de pathologie du grêle.

## Méthodes

**Type et lieu d´étude**: il s´agit d´une étude rétrospective descriptive et analytique s´étalant sur 8 ans entre janvier 2011 et juin 2019 réalisée au sein du service de gastro-entérologie de l´hôpital militaire et d´instruction Mohamed V de Rabat.

**Population d´étude**: elle a concerné au total cinquante-un (51) patients dont 30 hommes et 21 femmes d´âge moyen 48 ans. Les critères d´inclusion étaient: être atteint ou suspecté d´une pathologie du grêle soit à l´imagerie soit à la vidéo-capsule-endoscopique et avoir bénéficié d´une entéroscopie simple ballon .Les variables étudiées étaient la faisabilité technique du geste, ainsi que ses indications, ses résultats et ses complications.

**Collecte des données**: les données ont été collectées à partir des dossiers des patients. Dans un second temps, le logiciel SPSS a été utilisé pour les analyses statistiques. Le traitement des données s´est fait en pourcentage, en moyenne ou en médiane. Le recueil des données a été effectué avec respect de l´anonymat des patientes et de la confidentialité de leurs informations. Une analyse descriptive a ensuite été réalisée.

**Considérations éthiques**: les informations ont été recueillies après consentement éclairé des patients et avec l´accord de la direction médicale scientifique de l´hôpital militaire et d´instruction Mohamed V de Rabat.

## Résultats

**Données socio-démographiques**: cinquante-un (51) patients ont bénéficié d´une entéroscopie au sein de notre service durant cette période. Il y´avait 30 hommes (59%) et 21 femmes (41%). L´âge moyen était de 48 ans (18 ans-91 ans).

**Données sur faisabilité du geste**: la technique adoptée chez tous les patients était une entéroscopie simple ballon sous anesthésie générale avec intubation des voies respiratoires. Les patients avaient observé un jeun de 6H pour l´entéroscopie haute, un régime sans résidus de 3 jours avec 4 litres de polyéthylène glycol (PEG) le soir de la veille de l´examen pour l´entéroscopie basse, et combiner les deux préparations en cas d´entéroscopie haute et basse. La préparation était bonne chez tous les patients. La durée de l´examen était en moyenne de 45 min par voie haute et de 60 min par voie basse. Le grêle a été exploré jusqu´à l´iléon proximal en cas d´entéroscopie haute et jusqu´au-delà de 120cm de la DAI en cas d´entéroscopie basse.

**Figure 2:**
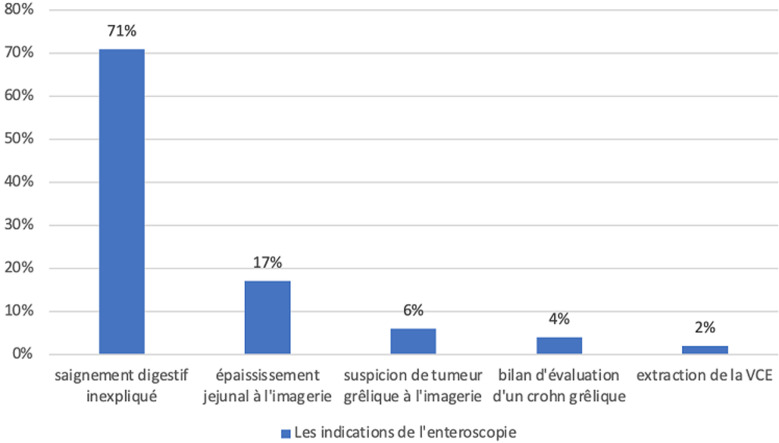
graphe illustrant les différentes indications de l´entéroscopie simple ballon dans le service de gastro-entérologie à l´hôpital militaire et d´Instruction Mohamed V de Rabat

**Données sur les indications de l´entéroscopie**: comme résumé dans la Figure 2, les indications étaient: par voie haute on dénombre 45 patients qui présentaient des saignements digestifs inexpliqués (n=33), un épaississement jéjunal a l´imagerie (n=8), une maladie de Crohn (n=1) et pour suspicion de tumeur du grêle à l´imagerie (n=3). Par voie basse on dénombre 4 patients qui présentaient un saignement digestif inexpliqué (n=2), une maladie de Crohn (n=1) et pour retrait de la VCE (n=1). Enfin, 2 patients ont bénéficié à la fois d´une entéroscopie haute et basse pour des saignements digestifs inexpliqués.

**Figure 3:**
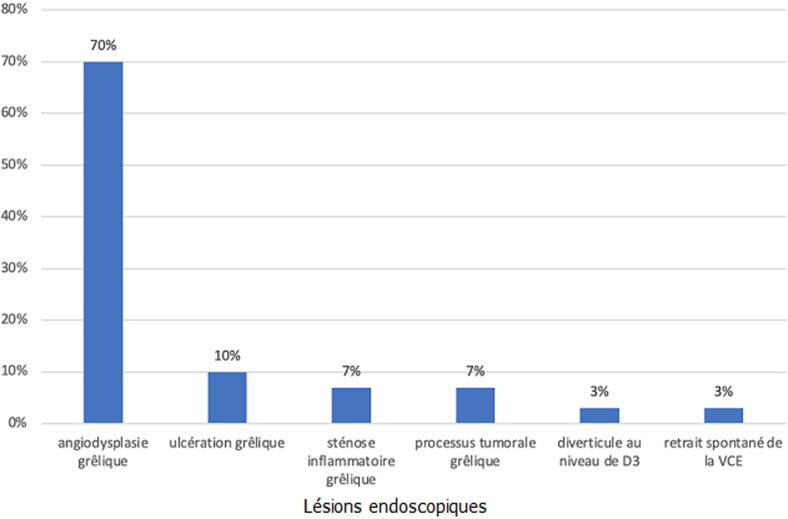
graphe illustrant les lésions endoscopiques retrouvées à l´entéroscopie simple ballon dans notre étude à l´hôpital militaire et d´Instruction Mohamed V de Rabat

**Données sur les résultats et complications de l´entéroscopie**: un diagnostic a été réalisé ou confirmé chez 29 patients soit une rentabilité diagnostic de 57%: les résultats sont répertoriés dans la Figure 3. Les angiodysplasies dominaient le classement des résultats et représentaient 70% des lésions retrouvés. Par conséquent cela a conduit à une électrocoagulation au plasma argon chez 20 patients soit une rentabilité thérapeutique de 39%. Aucune complication n´a été observée, l´exploration du grêle n´a pas été possible uniquement par voie basse chez 2 patients par échec de cathétérisme de la dernière anse iléale.

## Discussion

L´objectif de cette étude était de décrire la faisabilité technique de l´entéroscopie simple ballon mais aussi sa tolérance, ses indications et ses résultats obtenus dans un groupe de patients atteints ou suspects de pathologie du grêle. Cette présente étude montre que l´entéroscopie simple ballon reste bien tolérée et permet d´explorer une grande longueur du grêle. Les indications étaient variées et dominées par les saignements digestifs inexpliqués. La rentabilité diagnostique était de 57% et la rentabilité thérapeutique de 39%. Les limites de cette étude sont liées essentiellement au nombre réduit de patients inclus, (51 patients) et à son caractère monocentrique réalisé uniquement dans un seul centre. Toutefois, ces résultats pourraient constituer une base de données pouvant enrichir davantage la bibliographie et servir de référence pour les futurs travaux sur l´entéroscopie simple ballon. L´entéroscopie simple ballon est de fabrication japonaise, d´une firme appelée Olympus, et consiste à utiliser un surtube flexible à usage unique surmonté d´un ballon et un entéroscope sans ballon. Le principe est identique à celui de l´entéroscopie double ballon mais pour « accrocher » l´intestin grêle lors de la phase de redressement, on utilise le béquillage forcé associé au ballon du surtube [4].

La préparation de l´entéroscopie haute est la même que celle de la gastroscopie. Pour que l´examen se passe dans de bonnes conditions, il faut que l´estomac soit vide. Il ne faut donc ni boire, ni manger durant les 6 heures précédant l´examen, retirer les fausses dents mobiles si le patient en possède. La préparation de l´entéroscopie basse est la même que celle de la coloscopie et consiste donc à suivre un régime sans résidus dans les 3 jours qui précèdent l´examen associé à la prise 4 litres de polyéthylène glycol (PEG) le soir la veille de l´examen. Dans notre étude, l´indication prédominante et de loin était le saignement digestif inexpliqué. Les angiodysplasies en étaient la 1^ère^ cause [5] soit 70% des lésions retrouvés et ils étaient observés dans la population adulte [6] avec un âge moyen de 69 ans et des limites d´âges allant de 52 ans à 91 ans. Par ailleurs, on observe entre 30 et 50 ans, que les tumeurs (léiomyomes, tumeurs carcinoïdes, lymphomes et adénocarcinomes) sont prédominantes [7]. Chez les malades plus jeunes, le diverticule de Meckel est l´étiologie la plus fréquente. Outre les patients suivis pour une maladie de Crohn chez qui l´examen a objectivé une maladie toujours active, 3 cas d´ulcérations et 2 cas de sténose grêlique d´allures inflammatoires étaient également découvert dans le groupe des saignements inexpliqué Kwo et Tremaine [8] ont observés des resultats similaires et incriminaient la prise d´anti-inflammatoire non stéroïdiens dans leur étude.

La rentabilité diagnostique de l´entéroscopie est bonne, entre 60 et 80% selon les études.la rentabilité thérapeutique varie de 40 à 70%. Dans notre étude, elles étaient respectivement de 57% et de 39%. Les différentes techniques d´entéroscopies sont comparables en termes de rentabilité diagnostique et thérapeutique, de longueur de grêle exploré et de durée d´examen [9-11]. Il n´a pas été observé de complication dans notre étude, de même dans la littérature elles sont exceptionnelles et représentées par Les perforations digestives qui surviennent chez des patients ayant une pathologie chronique intestinale avec une paroi fragile ou suite à une polypectomie. Les hémorragies post-polypectomies (5 à 10% des cas) sont dans la grande majorité des cas traités pendant la procédure avec des clips hémostatiques et/ou des injections d´adrénaline diluée. Le risque de pancréatite aiguë reste faible (moins de 0,5%) et est surtout observé avec l´usage de l´entéroscopie double ballon du fait de l´hyperpression duodénale intéressant la région papillaire [12]. Le dosage de la lipasémie ne doit pas être systématique mais motivé par des douleurs abdominales de type pancréatique après la procédure. Des effets secondaires mineures (douleurs oropharyngées ou abdominales, pic thermique ou vomissement) ont été observés chez environ 25% des patients. Les douleurs abdominales après l´entéroscopie ont une évolution rapidement favorable dans la grande majorité des cas et l´utilisation d´un insufflateur à CO_2_, permet d´avoir une diminution de ce type de complication [13].

## Conclusion

**Figure 4:**
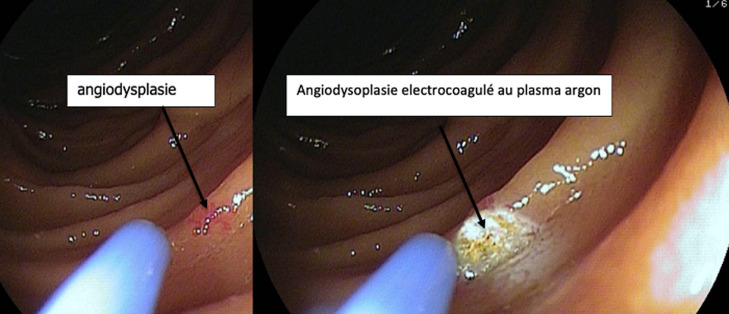
traitement des lésions d´angiodysplasie grêlique par électrocoagulation au plasma argon

L´entéroscopie est un examen a visé diagnostic et surtout thérapeutique dans l´exploration de l´intestin grêle. Dans notre étude la technique utilisée était l´entéroscopie simple ballon qui reste bien tolérée, et permet d´explorer une grande longueur du grêle. Les indications étaient variées mais dominées par les saignements digestifs inexpliqués. La rentabilité diagnostique était de 57% et la rentabilité thérapeutique de 39% essentiellement en rapport avec l´électrocoagulation au plasma argon des lésions d´angiodysplasie grêlique comme illustrée sur la Figure 4.

### Etat des connaissances sur le sujet

- Entéroscopie simple ballon est une technique d´exploration de l´intestin grêle utilisant un surtube;
- L´intérêt diagnostic: contrairement aux autres techniques d´exploration du grêle notamment radiologique (entéro-IRM et entéro-scanner) et la vidéocapsule endoscopique du grêle, l´entéroscopie utilisant un surtube est la seule technique permettant de poser le diagnostic des pathologies tumorales du grêle grâce à la réalisation de biopsie;
- L´intérêt thérapeutique: seule technique permettant également le traitement des pathologies du grêle (électrocoagulation des lésions d´angiodysplasie, dilatation de sténose grêlique…).

### Contribution de notre étude à la connaissance

- La rentabilité diagnostic de l´entéroscopie simple ballon était de 57% dans notre étude;
- La rentabilité thérapeutique de l´entéroscopie simple ballon était de 39% dans notre étude;
- Encourager les endoscopistes à s´y initier vu ses indications nombreuses et ses bonnes rentabilités diagnostic et thérapeutique.
